# Technical note: examining the response of a large carnivore (*Panthera leo*) to dummy prey

**DOI:** 10.1007/s10344-026-02135-2

**Published:** 2026-08-08

**Authors:** Anna Rouviere, Julius Muge, Graeme D. Ruxton, Robert A. Montgomery

**Affiliations:** 1 Department of Biology, University of Oxford, Oxford, UK; 2 Department of Zoology, Makerere University, Kampala, Uganda; 3 School of Biology, University of St Andrews, St Andrews, UK; 4 Institute of Science and Environment, University of Cumbria, Ambleside, UK

**Keywords:** Behaviour, Decoy, Fake prey, Field method, Model, Stalking

## Abstract

Animal dummies (sometimes called decoys or models) are widely used in ecological research to investigate predation, sexual selection, or social communication. The use of this method, however, is generally limited to small-bodied taxa, especially in predation studies. Yet, dummy prey could prove useful in understanding large carnivore behaviour, especially in situations where real-time observations and experimental approaches may be difficult. It is not known whether and to what extent large carnivores will recognise dummy prey as realistic in field experiments, which may hinder the development and funding of research using this method. In this study, we presented African lions (*Panthera leo*) with dummies visually resembling two types of prey in Murchison Falls National Park, Uganda. Lions showed predatory intent by stalking a dummy in 32% of all visits (*N* = 7 of 22), in 50% of first-time visits (*N* = 6 of 12), and in 63% of first-time visits involving a lone lion (*N* = 5 of 8). Based on our results, we suggest that dummy prey could be a useful tool in the study of the initial stages of a predation event, where a predator approaches its prey. We further discuss opportunities for, as well as potential limitations to, dummy use in the study of predation by large carnivores. We hope that this technical note will facilitate future study design using this methodology and encourage further exploration of the method.

**Supplementary Information:**

The online version contains supplementary material available at https://doi.org/10.1007/s10344-026-02135-2.

## Introduction

Presenting animals with dummies (or decoys, models) representing conspecifics, competitors, prey, or predators allows researchers to engineer interactions that may otherwise be difficult to observe or come with confounding factors. As long as they elicit appropriate reactions, dummies are a powerful experimental tool in fieldwork (Bateman et al. 2017). Dummies have been used to study communication or sexual selection in birds, reptiles, or even large mammals (Mougeot et al. 2001; West and Packer 2002; Bulté et al. 2018). Similarly, they have been prevalent in predation research, with use of dummy predators to study anti-predatory behaviours in various prey (Randall and Stevens 1987; Stenhouse et al. 2005), or dummy prey, commonly invertebrates, to understand predator preferences, behaviour, or habitat selection (Bateman et al. 2017; Roslin et al. 2017).

While dummies representing vertebrate prey are also used, they rarely represent mammalian prey and are rarely used to study large predator behaviour (Rundus et al. 2007; Bateman et al. 2017; Salvidio et al. 2019; Taylor and Cox 2019). Predation attempts by large carnivores usually involve high degrees of prey selectivity and sensitivity to prey behaviour (Husseman et al. 2003; Hubel et al. 2016). It is unknown to what extent such behavioural complexity by large predators impedes dummies triggering predatory behaviours. Two recent studies described large carnivore reactions to dummy prey (Gable and Gable 2019; Boone et al. 2023). However, one only assessed predator response based on whether the dummy was touched, thus not differentiating between investigative and predatory behaviour (Boone et al. 2023), and the other only described one instance of a wolf attacking a 2-D cut-out of a white-tailed deer (Gable and Gable 2019).

Potential scepticism surrounding the likelihood of dummy prey eliciting predatory behaviours from large predators may be hindering the development of experimental field studies using dummies. Yet, this methodology could help address research questions aiming to advance both carnivore conservation efforts and knowledge of carnivore hunting behaviour, for example through the investigation of variation in predatory strategies depending on human indicators (e.g., voice playbacks), or the influence of various prey-related sensory cues (e.g., visual, olfactory, auditory) on predator hunts. In this note, we assess reactions of African lions (*Panthera leo*) to dummies visually resembling two types of prey: a hartebeest (*Alcelaphus buselaphus*) and a domestic Zebu cow (*Bos indicus*). We aim to determine whether and under what circumstances lions, known for their characteristic stalking approach behaviour towards prey, can behave with fake prey as they would with live prey.

## Methods

### Study site

We conducted our study in the 250km^2^ Nile Delta region of Murchison Falls National Park (MFNP), Uganda. This region of the park is mainly composed of grassland and bushland (Plumptre et al. 2015; Mudumba et al. 2021), and hosts the highest lion densities in MFNP with 14–16 individuals per 100km^2^ (Braczkowski et al. 2024a, b). MFNP experiences two rainy seasons (March-June, August-November) and temperatures spanning 22–28 °C throughout the year (Akisiimire et al. 2022). Carnivores include lions, leopards (*Panthera pardus*), spotted hyenas (*Crocuta crocuta*), and side-striped jackals (*Lupulella adusta*) (Plumptre et al. 2015).

### Data collection

**Fig. 1:**
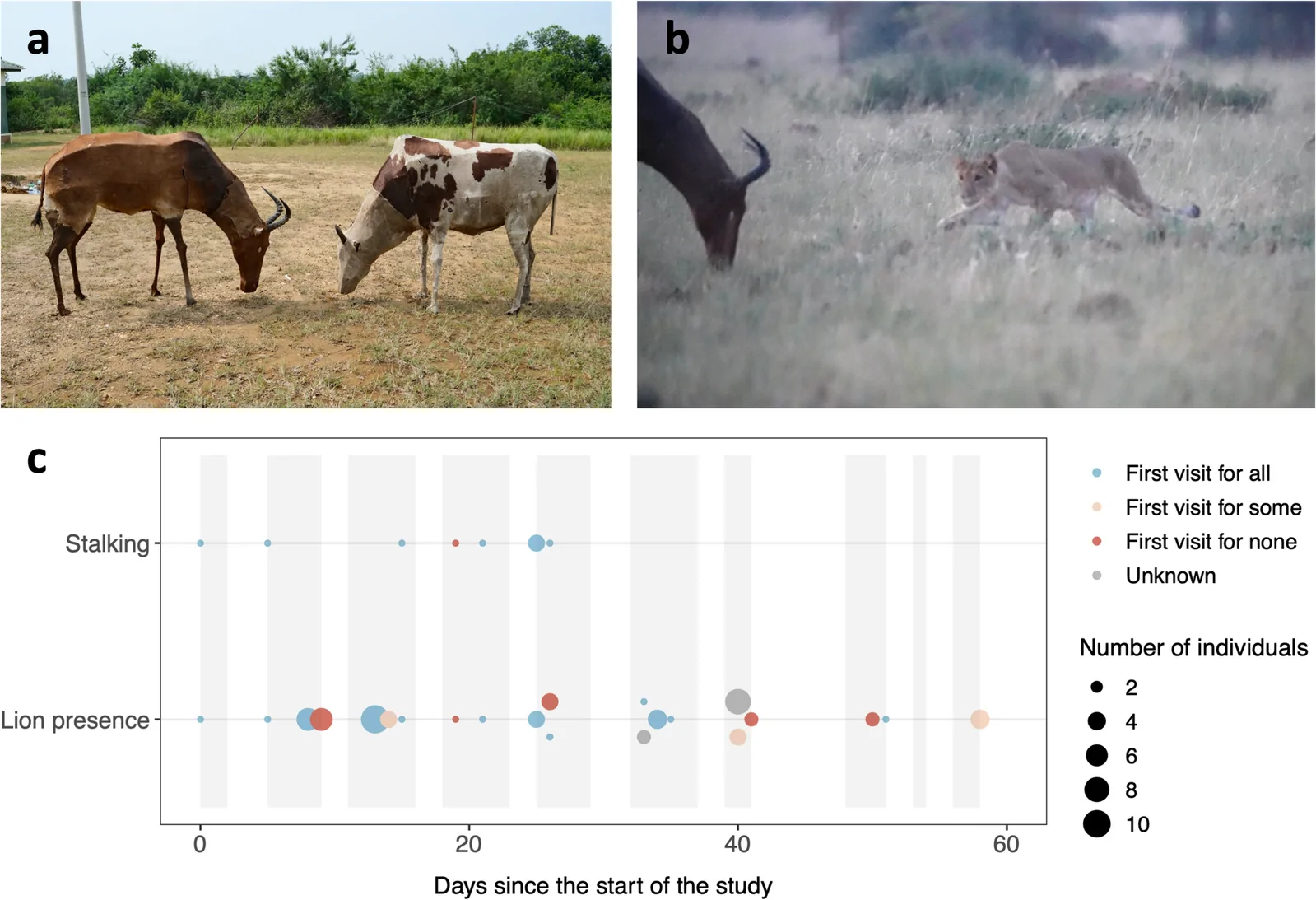
(**a**) Picture of the two life-sized dummy types used to assess lion reactions to dummy prey. (**b**) Photographic example showing one of the seven lions observed stalking dummy prey. The video footage from which this image was taken (Online Resource 4) was captured by Dylan Feldmeier. (**c**) Plot showing the timing, in number of days since the start of the study, at which all visits of lions to the experimental setup occurred (Lion presence), and all instances in which these visits led to a stalk of one dummy prey (Stalking). The areas shaded in grey indicate the days during which the experiment took place. Each point represents a distinct lion visit. The size of the point indicates the number of lions present, and the colour indicates whether all, some, or none of the lions were visiting for the first time. We could not assess this with certainty for two visits, as in each case we were not able to identify one lion present

We presented lions with life-sized dummies visually resembling two types of lion prey (Hayward and Kerley 2005; Olivier et al. 2023): hartebeest, common in MFNP (Rwetsiba and Nuwamanya 2010), and domestic Zebu cow, less likely to be encountered in this park. The dummies were built on site with a wire structure and covered with goat and cow hides (for the hartebeest and cow dummies respectively), these being available locally and ethically, and consistent with the target species’ colouration (Fig. 1a).

We conducted the experiment over 43 evenings from June to August 2024. Each evening, we placed one dummy of each type ~ 30 m apart in an open area. All dummy locations and a detailed description of our methods can be found in Online Resource 1. At around 19:00, we called lions, if none had been seen nearby, by playing distress calls – sounds that are not uncommon in the park – of prey distinct from our dummy species (warthog, duiker, buffalo) through a concealed speaker (Ailipu SP-3350D). If lions arrived, we recorded the time of arrival and number of lions, and filmed their interaction with the dummies. We identified individual lions using whisker patterns and recorded their sex and age class (Miller and Funston 2022). We ended the experiment at 21:00 each night.

To assess lion reactions to the dummies, we recorded the display of three types of behaviour: attention, inspection, and stalking. Attention was recorded if lion(s) focused their gaze in the direction of at least one dummy, no matter their distance to the dummy. Inspection occurred when lion(s) approached the dummies (not necessarily in a predatory manner) to within one body length and inspected them closely. This included sniffing or pawing, both of which are investigative behaviours in lions (Stanton et al. 2015). Finally, using the ethogram from Stanton et al. (2015) and our own observations, we defined a stalk as a slowed and directed movement towards one of the dummies, in conjunction with either a crouched body position or a noticeably exaggerated lifting of the paws during the approach. While attention and inspection could be investigative behaviours, stalking is strictly predatory in lions. Stalking has only ever been reported in the context of lion hunts and is included in various ethograms as a predatory approach behaviour (Scheel and Packer 1991; Macnulty et al. 2007; Stanton et al. 2015; Dunston et al. 2016). The slow speed at which this approach is conducted as well as the energetically demanding efforts displayed to remain undetected (i.e., crouching, exaggerated lifting of paws) are not shown in investigative or curious approaches of novel objects (Stanton et al. 2015).

## Results

Over the 43 evenings of the experiment, we recorded 22 instances of lion presence, nine of which involved a single lion (41%; Fig. 1c). We identified 35 individuals based on their whisker patterns but were unable to identify two. Of the identified lions, 19 (54%) were adults, including four males and 15 females, and 16 (46%) were subadults (in this case, any stage before adulthood), including eight males, six females, and two whose sex we could not positively confirm. We estimated that the lions were distributed among three prides. Online Resources 2 and 3 present the data associated with each evening of the experiment, and a dataset of identified individuals.

In 95% of cases (*N* = 21 of 22), at least one lion paid attention to the dummies. In 91% (*N* = 20 of 22), an inspection occurred. Finally, in 32% (*N* = 7 of 22), one lion was recorded to stalk one of the dummies, though none made predatory contact after stalking (Fig. 1b-c). Stalking behaviour was stopped within around 1–5 m of the dummies, likely when the lions realised that the prey was not real. Videos of stalks can be seen in Online Resources 4–9.

Among the seven stalking lions, five were adults, of which all were female, and two were subadults, including one male and one female. Six were alone during the stalk, while the seventh was a subadult in company of her mother and a sibling (Fig. 1b-c). Six lions conducted their stalk during their first visit to the experiment (Fig. 1c). One adult female had already visited once as part of a group of 10 but had not stalked then. Finally, five of the lions stalked the dummy resembling a hartebeest, while two stalked the one resembling a cow. None stalked both dummies during the same visit. The average time elapsed between the beginning and end of the stalking behaviour was 109 s (SD = 89s, *N* = 7, min = 5s, max = 240s).

Though lion presence occurred consistently throughout the study, all stalks occurred roughly within the first half of the experiment. The last stalk took place on the 22nd evening of the experiment (51%; Fig. 1c). All lone lions that had visited before then had stalked a dummy. After that point, however, three new lone lions visited but did not stalk (Fig. 1c).

## Discussion

We found that lions acted in a distinctly predatory manner by stalking dummy prey in one third of visits, half of first-time visits, and two thirds of first-time visits involving a lone lion. We therefore suggest that it is possible for large carnivores such as lions to perceive dummy prey to be real, and that dummy prey have potential to be used to study some aspects of predation behaviour in lions.

Our results showed that stalks were mostly performed by lone lions. In open landscapes such as ours, it may be that lions in groups did not stalk, not because the dummies did not appear convincing, but rather because a stealth-based predatory strategy may not be adapted to a group’s conspicuous appearance. More densely vegetated areas may provide better stalking opportunities for groups (van Orsdol 1984; Davies et al. 2016).

Our finding that seven lions stalked dummy prey, along with the fact that stalking requires a highly focused gaze towards a target prey, suggests that these lions were likely convinced by their visual resemblance to live prey. Although criticism of dummy use includes lack of movement of dummies, we suggest that predators relying on stealth to approach their prey, such as lions, may not be surprised by the static nature of dummies during their initial approach, as this may simply indicate to the predator that it has not yet been seen. This may have been facilitated by the head-down, grazing position in which we built the dummies. However, a likely limitation of static dummy use in a predation context arises once the predator has approached. In our study, no lions physically attacked the dummies, and predatory behaviour ended shortly before the dummies were reached (1–5 m). Therefore, any behavioural study of lions using static dummy prey would likely have to focus only on the initial phase of a predation event (thus precluding the study of bite locations and subjugation strategies, for example). It follows that large coursing predators may be less suited to static dummy use than stealth-based predators, as the former likely expect movement from their prey (Smith and Holekamp 2023). Opportunistic observations of spotted hyenas near the dummies in our study showed rapid and erratic movements which could not be identified as distinctly predatory.

Though our recommendation that dummies seem more suited for study of the initial rather than terminal phases of predatory interactions may appear limiting, plenty of research opportunities fulfil this condition. These can include experiments assessing prey choice, reactions to novel prey, or variation in approach strategies based on factors such as the presence of anthropogenic cues (e.g., human voice playbacks) near dummies. Additionally, it may be interesting, moving forward, to experiment with dummy prey of varying complexity to assess the importance and role of various sensory cues on carnivore hunts. This can involve, for example, adding sound or movement, or manipulating the nature or intensity of smells emanating from dummies. This may reveal either the minimum conditions needed to trigger stalking behaviour in lions, as observed in this study, or the point at which multisensory complexity becomes essential to the progression of a stalk into an attack, if at all.

An element of habituation may have also influenced stalking behaviour. Almost half of lion visits involved lions that had seen the dummies previously. Furthermore, though all lone lions that visited the dummies in the first half of the study carried out a stalk, no stalks occurred in the second half despite new lone lions visiting. We suggest that these new lions were likely members of one of the three prides known to us in the area and may have been nearby when other pride members visited the dummies. However, given our limited sample size, we cannot say with certainty that habituation drove this temporal change. Despite this, we recommend that studies making use of dummy prey be conducted in areas with larger carnivore populations to limit habituation risk. We also recommend varying dummy models to further limit this risk.

The strength of dummy use lies in its nature as an experimental tool, allowing researchers to control the conditions in which it is presented to target animals, and to separate potential confounding effects. Though we have identified limitations to dummy use in predation research involving large-bodied prey and predators, we show that expanding its use to these taxa is possible, and holds potential for future research.

## Supplementary Information

Below is the link to the electronic supplementary material.

8.07 KB

Online Resource 3 (XLSX 10.6 KB)

Online Resource 2 (XLSX 15.4 KB)

Online Resource 7 (MP4 10.2 MB)

Online Resource 9 (MP4 26.1 MB)

Online Resource 8 (MP4 45.4 MB)

Online Resource 6 (MP4 53.3 MB)

Online Resource 5 (MP4 78.9 MB)

Online Resource 4 (MP4 188 MB)

Online Resource 1 (PDF 285 KB)

## Data Availability

All data associated with this study can be found in the Supplementary Information. This includes a detailed description of our methods (Online Resource 1), one spreadsheet presenting data associated with each of the evenings during which this study was conducted (Online Resource 2), one spreadsheet listing all identified lion individuals involved in the experiment (Online Resource 3), and six videos showing six of the seven stalks of dummy prey described in this study (Online Resources 4-9). The seventh stalk was witnessed but could not be filmed due to technical limitations.
