# Supplementary material for: Technical note: examining the response of a large carnivore (Panthera leo) to dummy prey
Source: Eur J Wildl Res. 2026 Aug 8;72(5):103. doi: 10.1007/s10344-026-02135-2 (PMC13451379; doi:10.1007/s10344-026-02135-2)
Supplement: Supplementary file 1 [file 10344_2026_2135_MOESM10_ESM.pdf]

Online Resource 1

Technical Note: Examining the response of a large carnivore (*Panthera leo*) to dummy prey

European Journal of Wildlife Research

Anna Rouviere, Julius Muge, Graeme D. Ruxton, Robert A. Montgomery

Corresponding author: Anna Rouviere, Department of Biology, University of Oxford, Oxford, UK; anna.rouviere@biology.ox.ac.uk

Complete Methods

Study Site

We conducted our study in Murchison Falls National Park (MFNP), in north-western Uganda. The 3900km^2 national park is the largest protected area in the country (Kasozi et al. 2022), and is bordered to the south by two protected areas, Karuma Wildlife Reserve and Bugungu Wildlife Reserve (Plumptre et al. 2015). MFNP is bisected by the Victoria Nile which flows 115km from east to west, separating the park into southern (~2220km^2) and northern (~1680km^2) banks. Broad habitat types include woodland, grassland, and bushland. Woodland is predominant in the southern bank and in the northern and eastern parts of the northern bank. The western part of the northern bank, however, which is the region of the Nile Delta, is mainly composed of grassland and bushland (Plumptre et al. 2015; Mudumba et al. 2021). MFNP experiences two rainy seasons throughout the year (March-June and August-November), and temperatures usually range between 22°C and 28°C year-round (Akisiimire et al. 2022). MFNP is home to a range of large mammals, including the largest remaining population of Rothschild's giraffe (*Giraffa camelopardalis rothschildi*) (Muller 2019), as well as African savanna elephants (*Loxodonta africana*), Cape buffalo (*Syncerus caffer caffer*), Ugandan kob (*Kobus kob thomasi*), oribi (*Ourebia ourebi*), hartebeest (*Alcelaphus buselaphus*), and warthog (*Phacochoerus africanus*) (Plumptre et al. 2015). Carnivores include lions, leopards (*Panthera pardus*; 14.06 individuals per 100km^2), spotted hyenas (*Crocuta crocuta*; 45.31 individuals per 100km^2), and side-striped jackals (*Lupulella adusta*) (Plumptre et al. 2015; Braczkowski et al. 2024a).

The lion population varies in density throughout MFNP. While on average, lion density has been reported at 7.4 individuals per 100 km^2, this is mostly driven by high densities in the Nile Delta region (between 14 and 16 individuals per 100 km^2) as numbers decline sharply in the rest of the park (Braczkowski et al. 2024a, b). For this reason and because of the presence of open habitats, we chose to specifically conduct our study within and around the 250 km^2 Nile Delta region, in the north-west of MFNP (Fig. S1). The specific locations at which we conducted our study were in the latitude range of 2.26216 to 2.45009, and in the longitude range of 31.39582 to 31.70241 (Fig. S1).

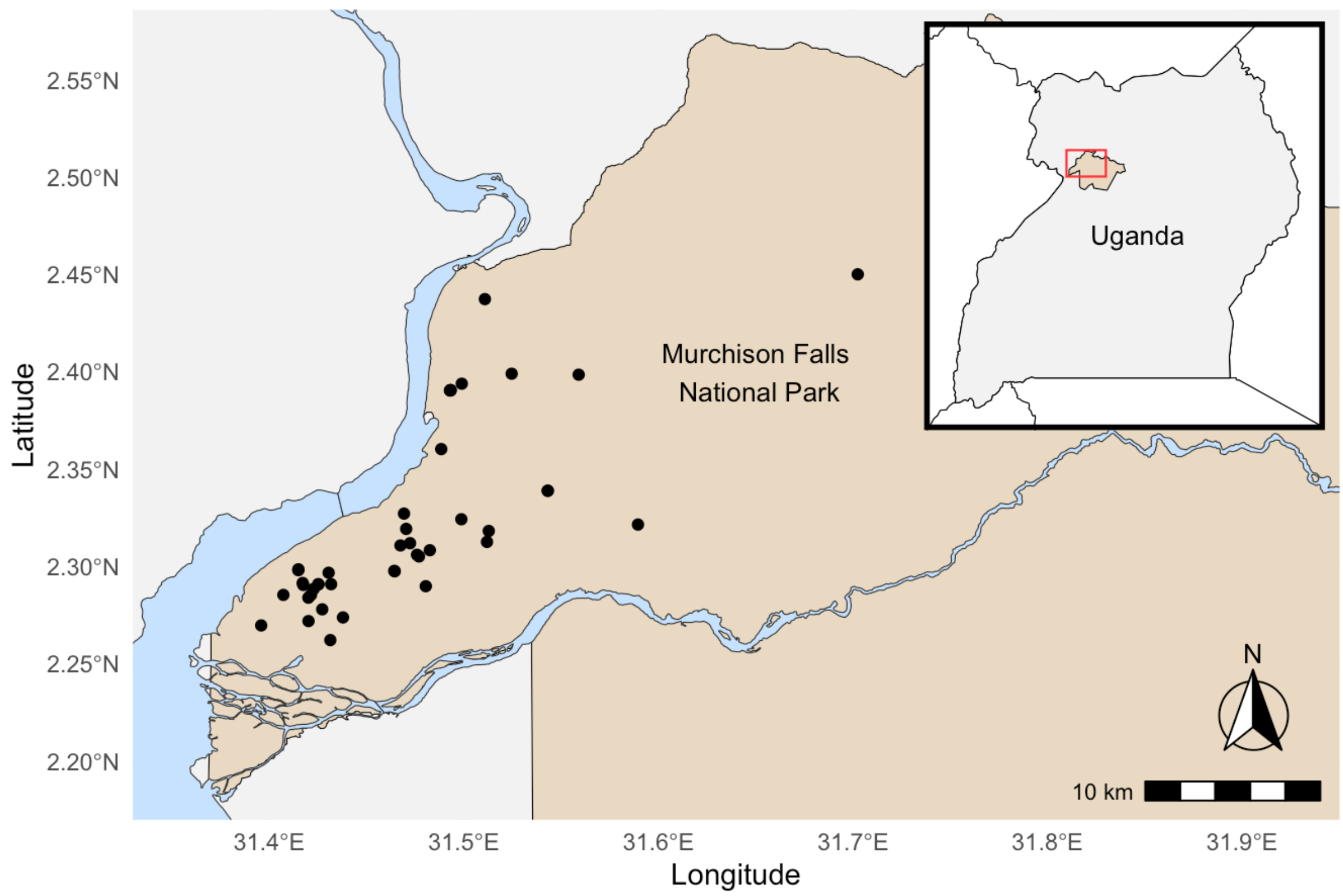

Fig. S1 Map showing the 43 locations at which prey dummies were placed within the Nile Delta region of Murchison Falls National Park (MFNP), Uganda, between June and August 2024. Black points represent the dummy locations, and the national park is shaded in tan. MFNP shapefile available from UNEP-WCMC & IUCN (2024)

Data Collection

To assess lion reactions to prey dummies, we chose to present lions with two different life-sized dummies representing two types of prey that lions are known to predate on. The first type was built to look like a hartebeest (*Alcelaphus buselaphus*), a wild prey species with an

estimated abundance of over 3500 individuals in MFNP and a prey of lions therein (Hayward and Kerley 2005; Rwetsiba and Nuwamanya 2010). The second type was built to look like a Zebu cow (*Bos indicus*), a domestic prey known to be predated on by lions throughout their range, but less likely to be encountered regularly by the lions in this park (Patterson et al. 2004; Kissui 2008; Olivier et al. 2023). Both dummy types were built in a head-down, grazing position. The dummies were built with a wire structure and covered with hides (Fig. 1a). The hides available locally and ethically were cow and goat, so the first dummy type was covered with goat hides consistent with hartebeest colouration, while the second dummy type was covered with cow hides. To minimise the presence of human markers on the dummies due to repeated manipulation, we regularly replaced both existing hides on our dummies with new sets of hides.

We conducted the experiment over 43 evenings between the 6th of June and the 3rd of August 2024. At around 16:00, we drove within the study area looking for lions, which at this time would still be resting. If we found lions, we drove to a nearby open area out of the line of sight of the lions to set up the dummies. If we did not find lions, we chose a suitable open habitat within the study site with no visual obstacles. We placed the two dummies 30m apart and facing each other. We concealed a speaker at the halfway point between them, usually within a small shrub. The locations of our dummies during each evening of the experiment can be seen in Fig. S1.

After placing the dummies, we retreated to a distance of ~75m away, to minimise any disturbance caused by our presence. At around 19:00, we began playing distress calls of potential lion prey species through the speaker to call lions to the location of the dummies. The distress calls were of prey taxa present in the park, but distinct from our dummy species. These included warthog, duiker (Cephalophinae), and buffalo. Sounds such as these are not uncommon in the park. When lions arrived at the experimental setup, we used a thermal camera (in darkness) or a regular camera (in daylight) to film the encounter between the lion(s) and the dummies. In each case, we recorded the time of arrival, the number of lions, and whether these were lions we had encountered before setting up the dummies and knew were nearby. We ended the experiment at 21:00 each night.

To assess the reaction of lions to the dummy prey, we recorded the display of three types of behaviour by the lion(s) in the vicinity of the dummies: attention, inspection, and stalking. We recorded the lion(s) as paying attention to the dummies if they were seen focusing

their gaze in the direction of at least one of the dummies. An inspection was recorded when the lion(s) walked to the dummies and were seen to inspect them closely, within one body length of the dummy. Finally, we recorded whether there was a stalk of a dummy. While attention to and inspection of the dummies could be seen as curiosity-driven or investigative behaviour, stalking is strictly a predatory behaviour in lions (Scheel and Packer 1991). We therefore considered a stalk of one or both dummies to be evidence of the focal lion(s) acting towards the dummies as they would real prey. Using the ethogram developed by Stanton et al. (2015) as well as our own observations, we defined a stalk as a slowed and directed movement towards one of the dummies, in conjunction with at least one of the following: a crouched body position or a noticeably exaggerated lifting of the paws during the approach (see Online Resources 6 and 7 for examples of lifted paws during stalking). If a lion stalked one of the dummies, we recorded which dummy was stalked, the duration of the stalk, and whether the lion then stalked the other dummy. After the lion(s) had finished interacting with the dummies, we identified the individuals present, whenever possible, by taking photographs of their whisker patterns on both sides, and we made note of their sex and whether we considered them to be an adult or subadult (hereafter, the term 'subadult' includes any stage before adulthood; i.e., cubs, juveniles, and subadults). The latter was determined by a combination of visual factors, including whether they were independent of their mother, size, nose colouration, and appearance of the mane in males (Whitman et al. 2004; Miller and Funston 2022).

This study was conducted under a research permit granted by the Uganda Wildlife Authority (ref: COD/96 /05). The methods used for this study were reviewed and approved by the Biology Animal Welfare and Ethical Review Board at the University of Oxford (ref: APA/1/5/ZOO/NASPA/Montgomerie/Hyena2024). The methods used were non-invasive, and the animals involved were not handled or harmed in any way. All individuals were fully free to approach or leave the experimental setup at any time. This study was conducted in accordance with the STRANGE framework (Webster and Rutz 2020). All individual lions identified during the study are listed, as well as their sex, age category, and social links, in Online Resource 3. A range of individuals, of both sexes and all age categories, participated in this study. Selection bias is most likely limited given that, based on recent density estimates (Braczkowski et al. 2024a, b), most if not all lion individuals present in our study area participated in the experiment (see Discussion section in manuscript for further details). Finally, potential effects of social background as well as acclimation and habituation are discussed in the third paragraph of the Discussion section. Though data for this study could not

be recorded blindly, recorder bias was minimised by classifying lion behaviours post-hoc, after carefully reviewing footage of lion-dummy interactions and comparing behaviours to the criteria listed above. One author (AR) was responsible for classifying behaviours, as both attention (which only involves a gaze towards the dummies) and inspection (based on physical proximity to within one body length of a dummy) were considered mostly unambiguous to classify. However, any behaviour that could be interpreted as stalking was checked by another person (Lara Boudinot, see acknowledgements). All stalks reported in this study were classified as such by both reviewers, and no disagreements occurred.

Additional Results

Lions that displayed inspection or stalking behaviours inevitably also displayed attention behaviour, which only requires the lion to look in the direction of a dummy. However, lions did not necessarily display all three behaviours concurrently. In one of the 22 encounter events between lions and the dummies, none of the three behaviours were shown. In one encounter event, only attention was displayed. In thirteen events, attention then inspection were shown. Finally, in seven events, attention was followed by stalking, which was followed by inspection.

References

Akisiimire H, Tinzaara W, Tumwebaze K, K.Twesigye C (2022) Assessment of vegetation in Murchison falls national park five years after the completion of oil and gas exploration. East African Journal of Biophysical and Computational Sciences 3:43–57. https://doi.org/10.4314/eajbcs.v3i1.5s

Braczkowski AR, Elliot N, Rwetsiba A, et al (2024a) Insights into Large Carnivore Populations in Uganda: A Participatory Survey of Lions, Leopards, and Hyenas Using Spatial Capture-Recapture. Glob Ecol Conserv e03312. https://doi.org/10.1016/j.gecco.2024.e03312

Braczkowski AR, Namukose L, Musobozi S, et al (2024b) Rangers on the frontline of wildlife monitoring: a case study on African lions in Uganda's Nile Delta. Commun Biol 7:1308. https://doi.org/10.1038/s42003-024-06796-0

Hayward MW, Kerley GIH (2005) Prey preferences of the lion (Panthera leo). J Zool 267:309–322. https://doi.org/10.1017/S0952836905007508

Kasozi H, Moll RJ, Kityo RM, Montgomery RA (2022) Phylogeny is a stronger predictor of activity than allometry in an African mammal community. Biological Journal of the Linnean Society 135:599–609

Kissui BM (2008) Livestock predation by lions, leopards, spotted hyenas, and their vulnerability to retaliatory killing in the Maasai steppe, Tanzania. Anim Conserv 11:422–432. https://doi.org/10.1111/j.1469-1795.2008.00199.x

Miller JRB, Funston P (2022) Aging the African Lion. http://agingtheafricanlion.com. Accessed 4 Dec 2024

Mudumba T, Jingo S, Heit D, Montgomery RA (2021) The landscape configuration and lethality of snare poaching of sympatric guilds of large carnivores and ungulates. Afr J Ecol 59:51–62. https://doi.org/10.1111/aje.12781

Muller Z (2019) Rothschild's giraffe Giraffa camelopardalis rothschildi (Linnaeus, 1758) in East Africa: A review of population trends, taxonomy and conservation status. Afr J Ecol 57:20–30. https://doi.org/10.1111/aje.12578

Olivier IR, Tambling CJ, Müller L, Radloff FGT (2023) Lion (Panthera leo) diet and cattle depredation on the Kuku Group Ranch Pastoralist area in southern Maasailand, Kenya. Wildlife Research 50:310–324. https://doi.org/10.1071/WR22019

Patterson BD, Kasiki SM, Selempo E, Kays RW (2004) Livestock predation by lions (Panthera leo) and other carnivores on ranches neighboring Tsavo National Parks, Kenya. Biol Conserv 119:507–516. https://doi.org/10.1016/j.biocon.2004.01.013

Plumptre AJ, Ayebare S, Mugabe H, et al (2015) Biodiversity surveys of Murchison Falls Protected Area

Rwetsiba A, Nuwamanya E (2010) Aerial surveys of Murchison Falls Protected Area, Uganda, March 2010. Pachyderm 47:118–123

Scheel D, Packer C (1991) Group hunting behaviour of lions: a search for cooperation. Anim Behav 41:697–709

Stanton LA, Sullivan MS, Fazio JM (2015) A standardized ethogram for the felidae: A tool for behavioral researchers. Appl Anim Behav Sci 173:3–16. https://doi.org/10.1016/j.applanim.2015.04.001

UNEP-WCMC, IUCN (2024) Protected Planet: The World Database on Protected Areas (WDPA) and World Database on Other Effective Area-based Conservation Measures (WD-OECM). www.protectedplanet.net. Accessed 5 Dec 2024

Webster MM, Rutz C (2020) How STRANGE are your study animals? Nature 582:337–340. https://doi.org/https://doi.org/10.1038/d41586-020-01751-5

Whitman KL, Starfield AM, Quadling HS, Packer C (2004) Sustainable trophy hunting of African lions. Nature 428:175–178. https://doi.org/10.1038/nature02395
